# Draft Genome Sequences of *Devosia* sp. Strain 17-2-E-8 and *Devosia riboflavina* Strain IFO13584

**DOI:** 10.1128/genomeA.00994-14

**Published:** 2014-10-02

**Authors:** Yousef I. Hassan, Dion Lepp, Jianwei He, Ting Zhou

**Affiliations:** a Guelph Food Research Centre, Agriculture and Agri-Food Canada, Guelph, Ontario, Canada; b School of Environmental Sciences, University of Guelph, Guelph, Ontario, Canada

## Abstract

Here we report the draft genome of *Devosia* sp. strain 17-2-E-8, isolated from Ontario agricultural soil (Canada) with promising deoxynivalenol biotransformation capabilities. In addition, we report the draft genome of *Devosia riboflavina* strain IFO13584, used as a control strain in our studies aimed at highlighting unique gene clusters involved in deoxynivalenol epimerization.

## GENOME ANNOUNCEMENT

*Devosia* as a genus has recently gained attention for the ability of many reported species to survive harsh environmental conditions, including hexachlorocyclohexane dump sites, diesel-contaminated soils, beach and deep-sea surface sediments, and alpine glacier cryoconite (1–12). Furthermore, a number of *Devosia* isolates have shown promising biodetoxification abilities that could lead to their utilization in agricultural and industrial applications as detoxifying agents (4, 13–16). Our laboratory recently reported the isolation of a new *Devosia* species, *Devosia* sp. strain 17-2-E-8 (IDAC 040408-1=ATCC PTA-121309) with a proposed designation of *Devosia epimeris*, which is capable of aerobically biotransforming deoxynivalenol (DON), a mycotoxin produced by various *Fusarium* species (15, 16). In our efforts to decipher the metabolic pathways responsible for this transformation, we report the *de novo* genome assemblies of two *Devosia* isolates: *Devosia* sp. strain 17-2-E-8 and *D. riboflavina* strain IFO13584 (ATCC 9526). *D. riboflavina* IFO13584 served as a control strain in these studies to highlight unique gene clusters in *Devosia* sp. 17-2-E-8 possibly involved in DON epimerization.

Genomic DNA from both isolates was purified using the Puregene Yeast/Bact. kit (Qiagen, Toronto, Canada) after 7 days of growth in LB broth and subjected to whole-genome sequencing using the MiSeq sequencer (Illumina, San Diego). Following fragmentation, end-repair, and sample indexing, a total of 17,343,120 and 15,745,680 of 300-bp paired-end reads were produced, resulting in 1,100× and 882× coverage for the *Devosia* sp. 17-2-E-8 and *D. riboflavina* IFO13584 genomes, respectively.

The FASTQC algorithm (17) was used to check the initial quality of our sequence reads. After removal of overrepresented sequences and quality trimming of the reads, *de novo* genome assemblies were generated using the CLC Genomics Workbench version 6.0.1 package. This resulted in a 4.7-Mb genome consisting of 125 contigs for *Devosia* sp. 17-2-E-8 and a 5.3-Mb genome consisting of 113 contigs for *D. riboflavina* IFO13584. Automated genome annotation was performed using both the RAST server (18) and the NCBI Prokaryotic Genome Annotation Pipeline (PGAP), which predicted 3,943 and 4,273 coding DNA sequences for *Devosia* sp. 17-2-E-8 and *D. riboflavina* IFO13584, respectively. An in-depth comparative genomic analysis of our data will be included in a future publication.

### Nucleotide sequence accession numbers.

Both whole-genome shotgun assemblies of *Devosia* sp. 17-2-E-8 and *D. riboflavina* IFO13584 were deposited at DDBJ/EMBL/GenBank under the accession numbers JQGB00000000 and JQGC00000000, respectively.
